# Single-cell transcriptome analysis reveals SOX9 mutation-driven tumor stemness and microenvironment remodeling through the COL1A1–CD44 axis in colorectal cancer

**DOI:** 10.1186/s12885-026-16248-z

**Published:** 2026-05-30

**Authors:** Yuwei Pan, Xuelian Xiang, Junfeng Li, Yinggang Ge, Xiaoyan Liang

**Affiliations:** 1 Department of Gastroenterology, The First Affiliated Hospital of Chongqing Medical University, Chongqing, 400016 China; 2 Department of Gastrointestinal Surgery, The First Affiliated Hospital of Chongqing Medical University, Chongqing, 400016 China

**Keywords:** Colorectal cancer, SOX9 mutation, COL1A1–CD44 axis, Tumor stemness, Single-cell RNA sequencing

## Abstract

Colorectal cancer (CRC) exhibits substantial interpatient heterogeneity and is driven by intricate crosstalk between tumor-intrinsic mutations and the tumor microenvironment (TME), yet the mechanisms by which specific genetic alterations remodel the TME and regulate tumor stemness remain elusive. The SRY-box transcription factor 9 (SOX9) is a key regulator of intestinal homeostasis, with mutations implicated in CRC progression, but its single-cell level effects on TME dynamics have not been fully elucidated. To dissect the heterogeneity and cell–cell interactions underlying SOX9 mutation-driven CRC progression, we integrated single-cell RNA sequencing (scRNA-seq) data of 23 CRC tumor samples with bulk sequencing and mutation data from The Cancer Genome Atlas (TCGA) using the Scissor algorithm. We identified a malignant epithelial subpopulation (Scissor^+^) specifically associated with SOX9 mutations, which exhibits enhanced stemness signatures. Further, we predicted a significant upregulation of the COL1A1–CD44 axis in SOX9-mutant tumors via CellChat, which was supported by immunohistochemical analyses in patient tissues and mouse xenograft models. By integrating stemness-related genes and fibroblast-derived ligands, we established a 172-gene fibroblast–stemness signature that was associated with overall survival, recurrence-free survival, and disease-specific survival across multiple independent CRC cohorts. Functional validation via CD44 knockdown and in vivo xenograft assays confirmed that the COL1A1–CD44 axis mediates SOX9 mutation-driven stemness maintenance and tumor progression. Collectively, our data enhance understanding of how SOX9 mutations remodel the TME, pinpoint the COL1A1–CD44 axis as a promising therapeutic candidate for SOX9-mutant CRC, and support precise patient stratification and targeted interventions.

**Supplementary Information:**

The online version contains supplementary material available at 10.1186/s12885-026-16248-z.

## Introduction

Colorectal cancer (CRC) represents a leading cause of cancer-related morbidity and mortality among gastrointestinal malignancies worldwide [1, 2]. While multimodal therapies incorporating surgery, chemoradiotherapy, targeted agents, and immunotherapy have substantially improved outcomes for early-stage disease, the 5-year survival rate for advanced CRC remains below 20%, with recurrence and distant metastasis representing critical barriers to therapeutic success [3, 4]. This clinical challenge stems fundamentally from the profound interpatient heterogeneity in tumor cell mutational landscapes, cancer stem cell properties, and dynamic interactions within the tumor microenvironment (TME), including immune and stromal compartments [5–7]. Bulk RNA sequencing (RNA-seq) analyses, by averaging signals across cellular populations, are inherently limited in resolving the cell type-specific molecular programs that drive CRC progression, necessitating single-cell resolution approaches to delineate these mechanisms.

The SRY-box transcription factor 9 (SOX9) is a pivotal regulator of intestinal development and epithelial homeostasis. In human CRC, SOX9 mutations occur in approximately 5–10% of cases, principally through uniparental disomy or missense alterations [8–10]. Under physiological circumstances, SOX9 governs intestinal stem cell fate through balanced self-renewal and differentiation, sustaining tissue integrity [11, 12]. Further, its dysregulated activation promotes oncogenic processes including proliferation, epithelial-mesenchymal transition, and stemness maintenance, with its overexpression correlating consistently with adverse prognosis in solid tumors such as hepatocellular carcinoma, breast cancer, and CRC [13–15]. However, prior studies have relied on cell line models or bulk RNA-seq data, leaving a gap regarding how SOX9 mutations sculpt the TME at single-cell resolution.

To address this, we leveraged the Scissor algorithm, a computational framework that integrates bulk phenotypes (e.g., mutation status) with single-cell RNA sequencing (scRNA-seq) to pinpoint phenotype-associated cell subpopulations [16]. By applying Scissor to scRNA-seq and bulk RNA-seq, we identified SOX9 mutation-linked cellular populations and dissected their TME remodeling properties through CellChat and gene set enrichment analysis. Furthermore, we derived a fibroblast-stemness signature and validated its prognostic utility across independent cohorts. Our study elucidates the mechanistic basis through which SOX9 mutations drive CRC progression via stromal reprogramming, providing a conceptual foundation for precision subtyping and therapeutic targeting in CRC.

## Materials and methods

### Data acquisition

ScRNA-seq data, comprising 23 CRC tumor samples, were downloaded from the Gene Expression Omnibus (GEO) database, specifically from series number GSE132465 [17]. Concurrently, bulk RNA-seq data and mutation data for CRC tissue samples were retrieved from The Cancer Genome Atlas (TCGA).

### ScRNA-seq data quality control

Quality control was implemented using R package Seurat v4.3.0 [18] with the following thresholds: (1) retaining cells expressing 200–6,000 genes; (2) excluding cells with < 1,000 unique molecular identifiers (UMIs); and (3) removing cells with mitochondrial gene content > 20%. The filtered cells were then subjected to downstream analyses.

### ScRNA-seq data dimension reduction, clustering and annotation

Following filtering, the gene expression matrices were normalized and transformed to the natural log scale. The FindVariableGenes function identified 2,000 highly variable genes for subsequent analyses. Batch correction across samples was conducted with Harmony algorithm (version 0.1.0) [19]. Principal component analysis (PCA) was carried out to reduce dimensionality using the highly variable genes. The first 15 dimensions were employed to cluster cells using the FindNeighbors and FindClusters functions (resolution = 0.1). Finally, the t-distributed stochastic neighbor embedding (t-SNE) method was applied to further reduce dimensionality based on the top 15 principal components and visualize the cells in a two-dimensional space. Cell types were annotated according to canonical markers. Feature and dot plots depicting marker expression for each cell type were generated using FeaturePlot and DotPlot functions.

### Scissor analysis

To identify cells associated with SOX9 mutation in scRNA-seq data, we collected gene mutation information from TCGA-COAD. Based on SOX9 gene mutation status, we divided CRC patients into SOX9-mutant and SOX9 wild-type groups, and compiled bulk RNA-seq and phenotype information. Subsequently, we analyzed the bulk and phenotype data with scRNA-seq data by running the R package Scissor [16] to estimate cell subpopulations with the highest mutation potential.

### Single-cell copy-number variant analysis

To confirm the malignant identity of epithelial cells, we inferred single-cell copy-number variation (CNV) profiles using the R package inferCNV (version 1.8.1). Raw count matrices of all epithelial cells were used as input, with plasma cells from immune populations serving as the reference normal population.

### Cell–cell communication analysis

Ligand–receptor (LR) interactions were predicted with CellChat (R package, v2.0.0) [20]. Separate CellChat objects were constructed for Scissor^+^ and Scissor^−^ cell subsets, and cellular crosstalk networks were compared between the two groups to identify differential cell–cell communications.

### xCell analysis

To characterize the cellular composition of the TME in SOX9 wild-type and SOX9-mutant patients from the TCGA-COAD cohort, we applied the xCell method implemented in the immunedeconv R package (version 2.1.0) [21]. This deconvolution approach assigns gene weights by quantifying the contribution of different cell types to specific gene expression profiles, allowing us to estimate fibroblast proportions and stromal scores.

### Gene set enrichment analysis

To assess the functional states of Scissor^+^ and Scissor^–^ tumor cells, we performed enrichment analysis with the R package fgsea (version 1.20.0) using 14 tumor-related functional states from CancerSEA (https://biocc.hrbmu.edu.cn/CancerSEA/), such as cell cycle, proliferation, and stemness, together with C8 cell-type gene sets from MSigDB (https://www.gsea-msigdb.org/gsea/msigdb) to identify potential differences in functional characteristics and cell type attributes. In addition, enrichment analysis was performed to compare Scissor^+^ and Scissor^−^ fibroblasts, with a focus on extracellular matrix remodeling and fibroblast activation-related pathways.

### Survival analyses with the gene expression signatures

By integrating the stemness-related gene set from CancerSEA (166 genes including SOX9, CD44) and differentially expressed fibroblast ligands predicted by CellChat (including COL1A1/2, COL6A1/2/3, FN1), we defined a 172-gene signature referred to as the fibroblast-stemness signature. The complete list of genes included in this signature is provided in Supplementary Table 2.

The association between this signature and patient survival was evaluated across five independent CRC cohorts (GSE17538, GSE29621, GSE38832, GSE87211 and GSE161158) obtained from the Gene Expression Omnibus (GEO, https://www.ncbi.nlm.nih.gov/geo/) database. Enrichment scores were calculated for each patient sample using the GSVA algorithm. Patients were stratified into high- and low-score groups using the median GSVA score as the cutoff within each cohort. The same cutoff strategy was applied uniformly across all survival analyses. Kaplan–Meier survival curves were plotted using the survival package (version 3.4.0) by integrating stratification information with clinical outcome data. Statistical significance was assessed using the log-rank test.

### H&E and immunohistochemical staining

Ten genetically-profiled treatment-naïve tissue samples were collected from the First Affiliated Hospital of Chongqing Medical University. Patients were stratified into SOX9 wild-type (*n* = 5) and SOX9-mutant (*n* = 5) cohorts based on genetic testing results. Additionally, SOX9 wild-type (WT) and SOX9-mutant (MUT) cell lines were subcutaneously implanted into nude mice at a dose of 8 × 10⁶ cells per mouse; tumors were harvested 18 days post-implantation. The study protocol was approved by the Institutional Review Board of the First Affiliated Hospital of Chongqing Medical University (approval number: 2025-639-01) and conducted in accordance with relevant ethical guidelines and regulations.

FFPE tumor sections (5 μm) of human tumor tissues and nude mouse-derived tumors underwent H&E and immunohistochemical staining. Following deparaffinization, rehydration, and peroxidase quenching (3% H₂O₂), antigen retrieval (citrate buffer, pH 6.0) and BSA blocking were performed. Primary antibodies were incubated overnight at 4 °C followed by HRP-conjugated secondary antibodies at 37 °C for 30 min. DAB development and hematoxylin counterstaining were applied before digital scanning with a KFBIO ScanScope. Antibodies were listed in Supplemental Table 1.

Semi-quantitative H-scores (0-300) were calculated for stromal COL1A1, tumor membranous CD44, and tumor nuclear SOX9 expression based on staining intensity (0 = negative, 1 = weak, 2 = moderate, 3 = strong) and positive percentage (0-100%) using the formula: H-score = (1×% weak) + (2×% moderate) + (3×% strong). All IHC scoring was performed in a blinded manner by two independent observers.

### Cell lines and cell culture

The HCT116 and LS180 cell lines used in this study were obtained from the cell bank of our laboratory. These cells were cultivated in HyClone DMEM (Gibco) medium, supplemented with 100 U/mL penicillin/streptomycin (Gibco) and 10% fetal bovine serum (FBS, ABW) to constitute the complete medium.

### Flow cytometry

For cell surface staining, two cell lines were harvested and resuspended in FACS buffer. Cell suspensions were stained using Fixable Viability Dye (eBioscience) to exclude dead cells, followed by incubation with anti-CD44 antibody (Biolegend) dilutions (4 °C, 30 min). All labeled cells were analyzed using a BD flow cytometry system with FlowJo software.

### Immunofluorescence

Two target cell lines were seeded onto coverslips and cultured in optimal growth medium to 50%–70% confluency. After medium aspiration, cells were incubated with Biotin-conjugated type I collagen (Biotin-COL1) at 4 °C for 1–2 h, followed by three washes with pre-cooled PBS (5 min each). Cells were fixed with 4% paraformaldehyde at room temperature for 20 min and washed three times with PBS. For blocking and permeabilization, cells were incubated with PBS containing 5% normal goat serum and 0.3% Triton X-100 at room temperature for 1 h. Subsequently, cells were incubated overnight at 4 °C in the dark with a mixture of anti-CD44 monoclonal antibody (clone G44-26, BD Biosciences) and anti-biotin antibody (1:100-1:500, per manufacturer’s instructions). After three PBS washes, cells were incubated with Alexa 488-conjugated donkey anti-mouse IgG (Invitrogen, 1:400) and Alexa 594-conjugated donkey anti-rabbit IgG (Invitrogen, 1:800) at room temperature for 1 h in the dark. Nuclei were counterstained with DAPI (Sigma, 10 µg·mL⁻¹) for 5 min in the dark, followed by three PBS washes. Coverslips were mounted with anti-fluorescence quenching medium, and fluorescent images were acquired using an Olympus fluorescence microscope.

### Tumorsphere formation assay

Cells were trypsinized to generate single-cell suspensions and seeded in 6-well plates (1 × 10^3^ cells/well) in DMEM/F12 medium containing 20 µl/ml B27, 20 ng/ml epidermal growth factor (EGF) and 20 ng/ml fibroblast growth factor (FGF). For COL1A1 treatment, hydrochloric acid-prepared COL1A1 (type I collagen, Sigma C3867) or vehicle control was added to the cell culture system every 3 days. Tumorsphere diameters were quantified after 10 days.

### Cell transfection and construction of stable cell lines

The shRNAs targeting CD44 (shCD44-1, 5’-CCGTTGGAAACATAACCATTA-3’; shCD44-2, 5’-GGACCAATTACCATAACTATT-3’; and shCD44-3, 5’-CGCTATGTCCAGAAAGGAGAA-3’) were acquired from Tsingke (Tsingke Biotechnology, China). Transfection was performed with Lipofectamine 2000 (Invitrogen, USA) following the manufacturer’s guidelines. Stable clones were screened for CD44 knockdown by Western blot, and the most effective shRNA was selected for further experiments.

### Western blot and Co-immunoprecipitation (Co-IP)

Cellular proteins were extracted in lysis buffer containing protease and phosphatase inhibitors (Beyotime, China) and quantified via BCA protein assay. Samples were separated by 10% SDS-PAGE and electrotransferred to PVDF membranes. The blots were blocked with 5% skim milk for 2 h, then probed with primary antibodies overnight at 4 °C. After primary antibody incubation, membranes were washed and incubated with HRP-conjugated secondary antibodies for 1 h. Protein bands were finally detected by enhanced chemiluminescence using a western blot detection kit (WBKLS0100, Merck Millipore, USA).

For the Co-IP assay, SOX9 MUT cells were treated with recombinant human COL1A1 protein. Cell lysates prepared with inhibitor-supplemented modified RIPA buffer were incubated with anti-CD44 or anti-COL1A1 antibody overnight at 4 °C. Immune complexes were captured using magnetic beads, followed by thorough washing. The eluted immunoprecipitates were analyzed by Western blot to assess the association between CD44 and COL1A1 under extracellular COL1A1 stimulation conditions.

### In Vivo tumor formation assays

Female BALB/c nude mice (6 weeks) from Beijing Huafukang Biotechnology were housed in a specific pathogen-free (SPF) environment. Cells were cultured to 70–80% confluence. For SOX9 WT and MUT tumor formation assays, cells were harvested, resuspended in serum-free DMEM at 8 × 10⁷ cells/mL, and 100 µL of cell suspension (8 × 10⁶ cells) was subcutaneously injected into the right axilla of BALB/c nude mice (*n* = 5 per group). Tumor volume (V = ab²/2, a=long diameter, b=short diameter) was measured every 3 days, and mice were euthanized by cervical dislocation on day 18. For SOX9 MUT co-culture assays, HCT116-SOX9 MUT cells stably transfected with shCtrl or shCD44 were treated with hydrochloric acid-prepared COL1A1 (type I collagen, Sigma C3867) for 24 h, while control groups received medium without COL1A1. Cells were harvested, resuspended in serum-free DMEM at 5 × 10⁷ cells/mL, and 100 µL of cell suspension (5 × 10⁶ cells) was subcutaneously injected into the right axilla of BALB/c nude mice (*n* = 5 per group). Tumor volume was measured every 3 days. On day 21, mice were euthanized by cervical dislocation.

### Statistical analysis methods

Statistical analyses were performed using R software version 4.2.3. Kaplan–Meier survival analysis was conducted using the survival package, and between-group comparisons were performed using the Log-rank test. For other comparisons, statistical significance was determined by unpaired Student’s t-test, one-way ANOVA or a two-way repeated-measures ANOVA. A P-value < 0.05 was considered statistically significant.

## Results

### The single-cell landscape of CRC patients

**Fig. 1:**
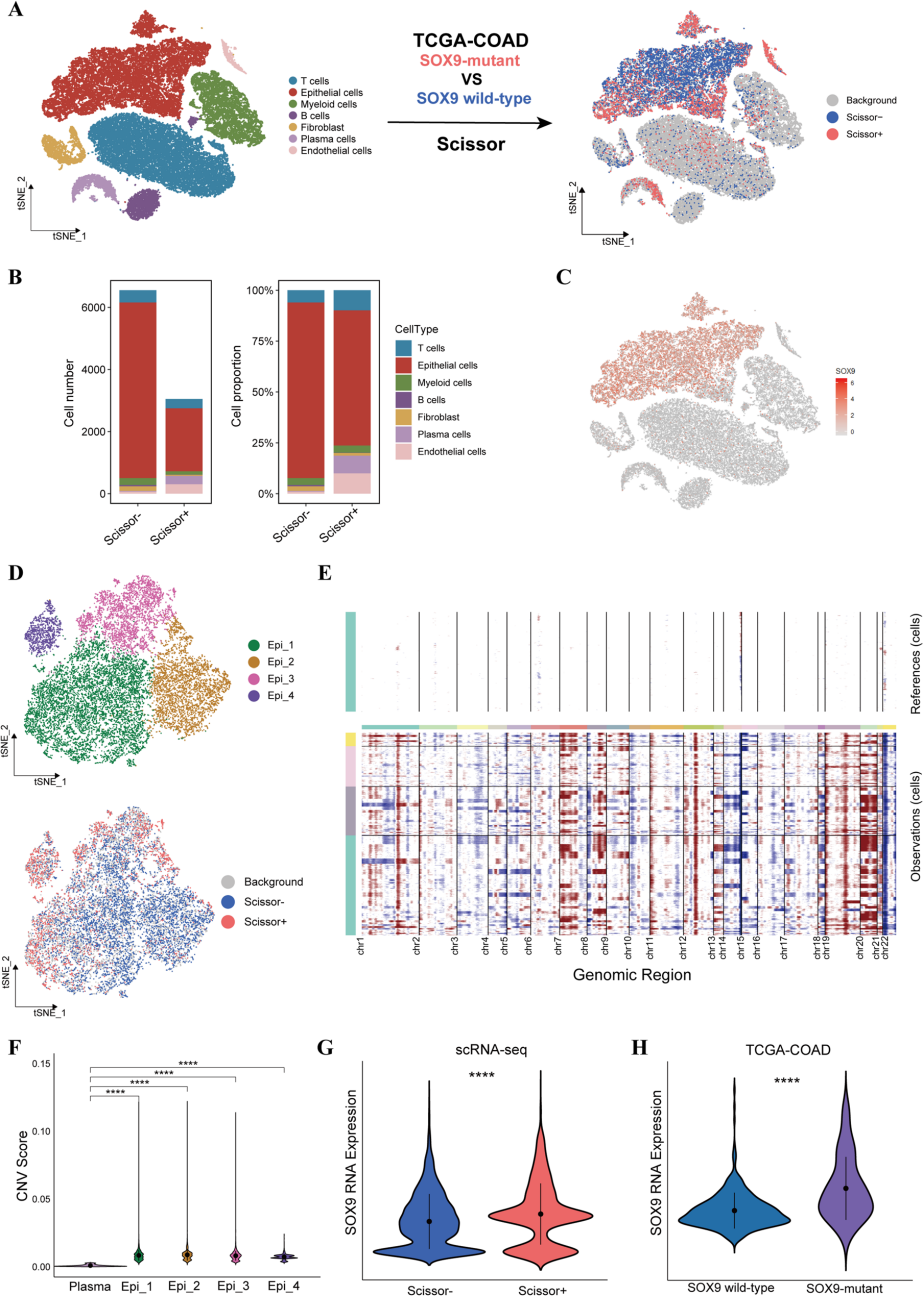
Identification of Scissor^+^ cells linked to SOX9 mutation. **A** Application of the Scissor algorithm to identify cell populations associated with SOX9 mutation status in the TCGA-COAD cohort. Left: t-SNE visualization of single-cell transcriptomic data colored by major cell types. Right: Scissor analysis results, showing cells positively correlated with SOX9-mutant tumors (Scissor^+^, red), negatively correlated with SOX9 wild-type tumors (Scissor^–^, blue), and background cells (gray). **B** Characterization of the cell type abundance and proportions identified in each group. **C** Feature plot presents the normalized expression levels of SOX9. **D** t-SNE maps of re-clustered epithelial cells and Scissor-defined groups. **E** Heatmap depicts Chromosome-wide CNV patterns across epithelial clusters. Red/blue indicate elevated and reduced CNV levels; plasma cells are the reference population. **F** Violin plot of CNV scores across epithelial clusters; Plasma box indicates reference cells. **G **Violin plot comparing SOX9 expression between Scissor^–^ and Scissor^+^ tumor cells (scRNA-seq). **H** Violin plot comparing SOX9 expression between SOX9 wild-type tumors and SOX9-mutant tumors (TCGA-COAD). An unpaired Student’s t-test was used to evaluate statistical significance (****: *P* < 0.0001)

Integration of scRNA-seq data from 23 CRC tumor samples (GSE132465) followed by stringent quality control yielded 45,285 high-quality cells. Graph-based unsupervised clustering via Seurat after normalization and dimensionality reduction identified seven cell subpopulations based on canonical marker expression: T cells (CD3D, CD3E, CCL5); epithelial cells (EPCAM, KRT8, KRT18); myeloid cells (LYZ, S100A8, S100A9); B cells (MS4A1, BANK1, CD79A); fibroblasts (DCN, LUM, COL1A1); plasma cells (IGHG1, MZB1, DERL3); and endothelial cells (PLVAP, PECAM1, VWF) (Fig. 1A, Supplementary Fig. 1A-B). Uniform cell type distributions across patients confirmed effective batch effect correction (Supplementary Fig. 1C). Moreover, detection of all seven cell types in each patient sample highlighted intratumoral heterogeneity and distinct cellular compositions (Supplementary Fig. 1D).

### Identification of Scissor^+^ cells linked to SOX9 mutation

Given the critical role of SOX9 mutation status in CRC, we employed Scissor, a supervised learning framework that maps bulk phenotypic labels onto single-cell transcriptomes, to transfer SOX9 mutation information from the TCGA-COAD dataset to our scRNA-seq data. This analysis identified cell subsets most strongly associated with SOX9-mutant phenotypes. Scissor identified 3,050 Scissor^+^ cells correlated with SOX9-mutant tumors and 6,554 Scissor^–^ cells associated with SOX9 wild-type tumors (Fig. 1A). Cell proportion analysis revealed that epithelial cells represented the dominant population among Scissor predicted cells and exhibited predominant SOX9 expression (Fig. 1B-C). Further, we re-clustered all epithelial cells, with Scissor predicted cells mapping across these clusters (Fig. 1D). To assess the malignant status of these epithelial cells, we applied the inferCNV algorithm using plasma cells as a reference. Relative to reference plasma cells, the four epithelial subpopulations exhibited significant chromosomal amplifications (chromosomes 7, 8, 12, 19, and 20) and deletions (chromosomes 14 and 22). CNV scoring confirmed significantly elevated CNV levels in all four epithelial subpopulations compared to plasma cells, establishing their malignant identity (Fig. 1E-F).

To validate these predictions, we compared SOX9 expression between Scissor^–^ and Scissor^+^ tumor cells. Scissor^+^ tumor cells displayed significantly elevated SOX9 expression compared to Scissor^–^ cells, matching the trend observed in bulk TCGA-COAD samples with SOX9 mutations (Fig. 1G-H). These findings demonstrate that Scissor successfully identifies SOX9 mutation-associated cell subpopulations, providing a basis for investigating the functional consequences of SOX9 alterations.

### SOX9 mutation remodels fibroblast–tumor cell communication and upregulates the COL1A1–CD44 ligand–receptor axis

**Fig. 2:**
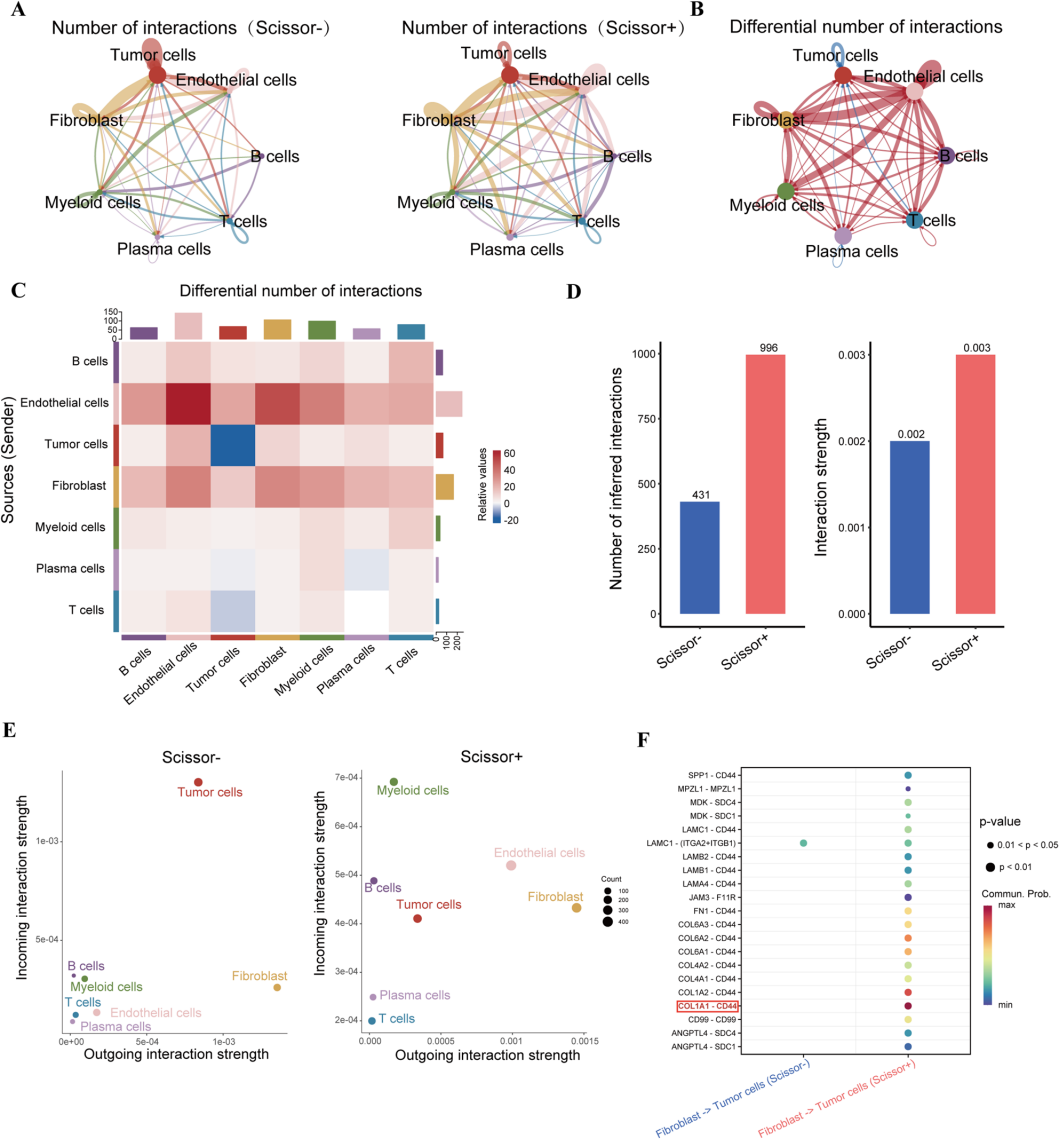
Analysis of distinct intercellular communication between Scissor^+^ and Scissor^–^ cells. **A** Cell–cell communication network among Scissor^–^ (left) and Scissor^+^ (right) cells. **B** Differential interaction network (Scissor^+^ vs. Scissor^–^). Red lines denote enhanced Scissor^+^ interactions. **C** Heatmap of cell–cell communication among annotated cell types, relative interactions strength between Scissor^–^ and Scissor^+^ cells. Red denotes enhanced Scissor^+^ interactions, while blue denotes enhanced Scissor^–^ interactions. **D** Bar plots comparing interaction counts and strength between Scissor^–^ and Scissor^+^ cells. **E** Outgoing interaction strength across cell types for Scissor^–^ (left) and Scissor^+^ (right) cells. **F** Dotplot of differential ligand–receptor interactions between Scissor^+^ and Scissor^–^ cells, where dot color indicates communication probability and dot size denotes statistical significance.

To dissect intercellular communication dynamics in the TME, we constructed CellChat-based networks for Scissor^–^ and Scissor^+^ cells, respectively (Fig. 2A). Scissor^+^ cells displayed significantly more extensive communication networks than Scissor^–^ cells (Fig. 2B-C). Quantitative analysis of global interaction number and strength confirmed elevated signaling activity in Scissor^+^ cells (Fig. 2D), indicating that SOX9 mutation enhances TME communication. Profiling outgoing versus incoming signals identified fibroblasts as the dominant source in both groups. However, their target cells differed: in the Scissor^–^ group, fibroblasts predominantly signaled to tumor cells, whereas in the Scissor^+^ group, they preferentially targeted myeloid cells, B cells, and tumor cells (Fig. 2E, Supplementary Fig. 2A). To further investigate key cellular communications between fibroblasts and tumor cells, significant changes in ligand–receptor pairs between the two groups of fibroblasts and tumor cells were compared. Notably, COL1A1–CD44, COL1A2–CD44, and FN1–CD44 pairs exhibited significantly enhanced intensity in the Scissor^+^ group, especially COL1A1–CD44 (Fig. 2F). In addition, among all significantly regulated ligand–receptor pairs acting on tumor cells as receptors, the COL1A1–CD44 signaling axis formed by interactions between fibroblasts and tumor cells exhibited the most pronounced differential expression and the highest communication probability (Supplementary Fig. 2B).

**Fig. 3:**
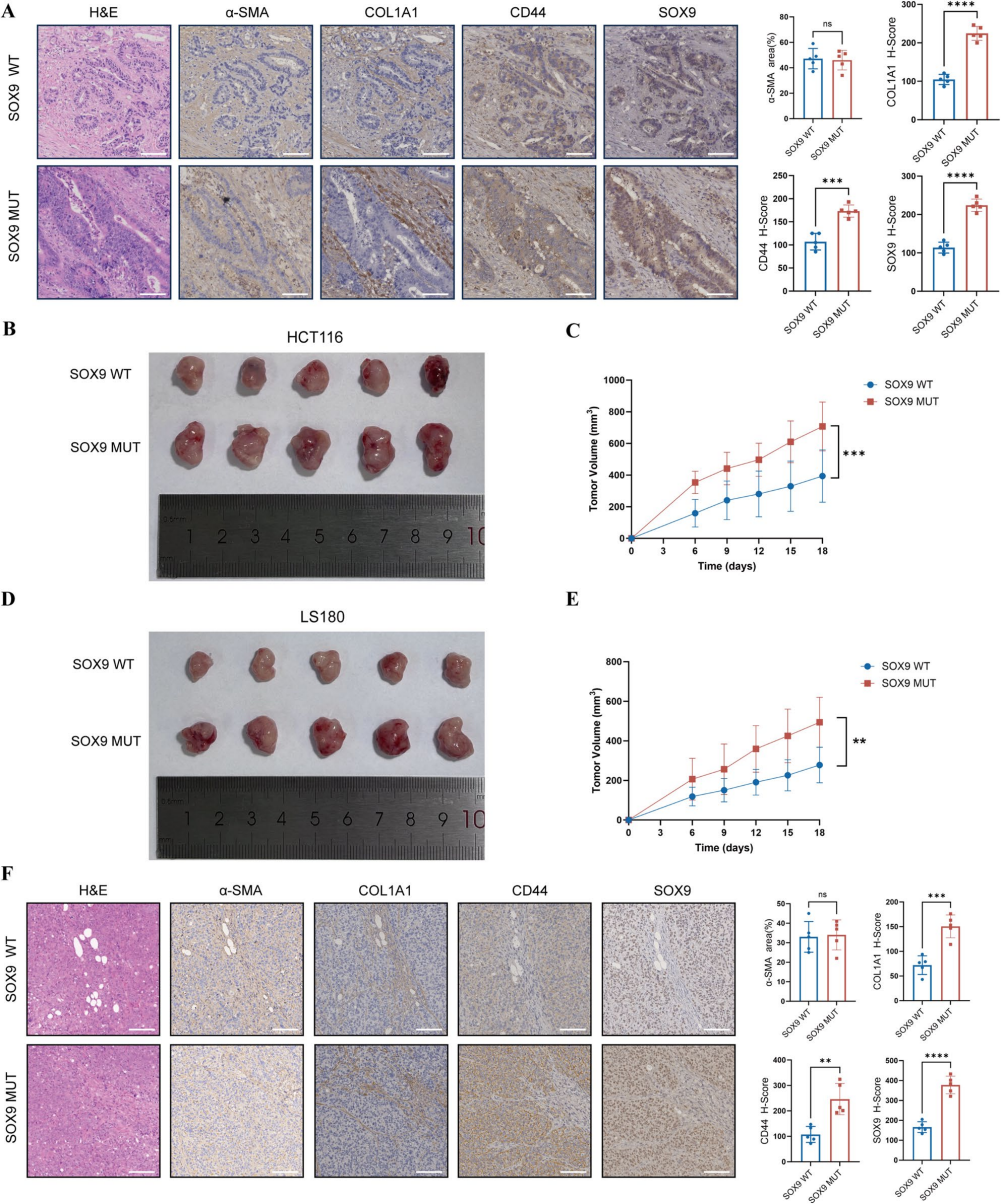
SOX9 mutation remodels fibroblast–tumor cell communication and upregulates the COL1A1–CD44 ligand–receptor axis. **A** Representative H&E staining and immunohistochemical analysis of α-SMA, COL1A1, CD44, SOX9 in CRC patients from SOX9 WT and SOX9 MUT tumors. Quantification of α-SMA-positive area and H-scores for COL1A1, CD44, and SOX9 are shown on the right. Scale bar: 200 μm. **B**, **D** Representative gross images of tumors excised from nude mice 18 days after subcutaneous implantation of HCT116 (**B**) or LS180 (**D**) cells with either SOX9 WT or SOX9 MUT status. **C**, **E** Tumor growth curves showing quantification of tumor volume in each group, measured every 3 days over a 18–day period for HCT116 (**C**) and LS180 (**E**) xenograft models. **F** Representative H&E staining and immunohistochemical analysis of α-SMA, COL1A1, CD44, and SOX9 in tumor tissues from the xenograft models with SOX9 WT and SOX9 MUT status. Quantification of α-SMA-positive area and H-scores for COL1A1, CD44, and SOX9 are shown on the right. Scale bar: 200 μm. **A**-**F** Data are presented as mean ± SEM (*n* = 5). An unpaired Student’s t-test or a two-way repeated-measures ANOVA followed by multiple comparisons tests was used to evaluate statistical significance (ns: *P* > 0.05; **: *P* < 0.01; ***: *P* < 0.001; ****: *P* < 0.0001).

To validate these bioinformatic findings in clinical samples, we next performed immunohistochemical analysis on primary CRC tissues from patients with known SOX9 mutation status. We collected tumor samples from CRC patients who had undergone genetic testing—both SOX9 wild-type and SOX9-mutant cases—and subjected them to immunohistochemical staining. COL1A1 is primarily secreted by cancer-associated fibroblasts (CAFs) in various cancers [22]. Immunohistochemical results revealed spatial overlap between α-SMA and COL1A1 staining. Notably, SOX9-mutant tumors exhibited stronger nuclear SOX9 and membranous CD44 signals in malignant cells, along with increased stromal COL1A1 deposition (Fig. 3A), whereas no significant difference in α-SMA-positive area was observed between the two groups.

Bioinformatic analysis of single-cell data and the TCGA-COAD cohort using the xCell algorithm further demonstrated that SOX9 mutation did not affect overall fibroblast abundance, as evidenced by comparable fibroblast proportions in Scissor⁻/Scissor⁺ subgroups, as well as similar fibroblast ratios and stromal scores between SOX9-mutant and wild-type patients in TCGA-COAD (Supplementary Fig. 3A-B). Differential gene analysis of Scissor⁻ versus Scissor⁺ fibroblasts revealed that Scissor⁺ fibroblasts exhibited elevated expression of extracellular matrix-related genes including COL1A1, FN1, and MMP14, accompanied by enrichment of key transcriptional regulators of collagen synthesis such as CREB3L1 and GLI2 [23, 24] (Supplementary Fig. 3C). Pathway enrichment analysis indicated significant activation of ECM–receptor interaction and TGF-β signaling pathways in Scissor⁺ fibroblasts (Supplementary Fig. 3D), both of which have been implicated in enhancing fibroblast collagen secretory function [25, 26]. Collectively, these bioinformatic results suggest that SOX9 mutation may reshape the phenotypic characteristics of fibroblasts in the TME and potentially enhance their collagen secretory capacity.

To further validate our findings in vivo, following the experimental strategy described in our previous study [14], we generated SOX9 truncation mutant (SOX9 MUT) lines from HCT116 and LS180 CRC cells, and further constructed subcutaneous xenograft models in nude mice. In vivo experiments demonstrated that SOX9 mutation significantly promoted CRC xenograft growth (Fig. 3B-E). Immunohistochemical analysis of xenograft tissues confirmed that α-SMA-positive area showed no significant difference between the two groups; however, nuclear SOX9 expression, membranous CD44 signals in tumor cells, and stromal COL1A1 deposition were markedly upregulated in the SOX9 MUT group (Fig. 3F), consistent with our findings from clinical samples and bioinformatic analyses.

Collectively, these results indicate that SOX9 mutation is associated with altered intercellular communication patterns within the TME, particularly in fibroblast–tumor cell interactions. Bioinformatic analyses and experimental findings suggest that fibroblasts in the context of SOX9 mutation may exhibit enhanced collagen-related secretory features, accompanied by elevated activity of ligand–receptor signaling axes such as COL1A1–CD44.

### Stemness-associated pathways and stem-like features in SOX9-mutant tumor cells

**Fig. 4:**
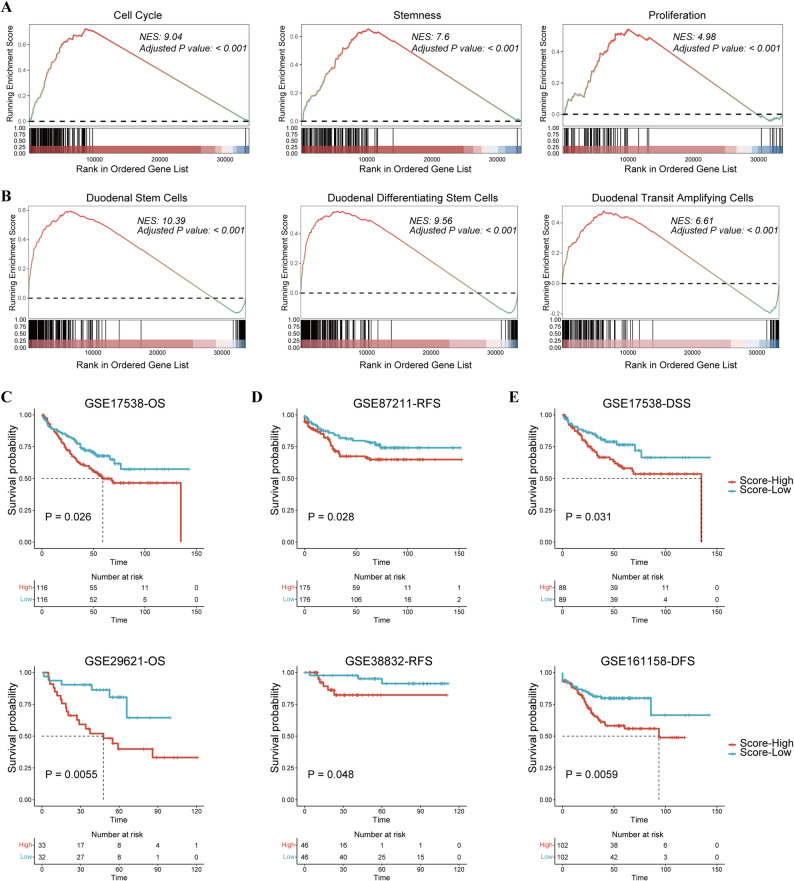
Stemness features in SOX9-mutant tumor cells and prognostic value of a fibroblast–stemness signature in CRC. **A** GSEA enrichment plots demonstrating significant upregulation of cell cycle, stemness, and proliferation pathways in Scissor^+^ tumor cells versus Scissor^–^ cells. All pathways showed adjusted *P*-values < 0.001. **B** Enrichment of intestinal stem cell signatures in Scissor^+^ tumor cells, including duodenal stem cell, duodenal differentiating stem cell, and duodenal transit-amplifying cells. **C-E** Kaplan–Meier survival curves comparing high-score and low-score patient groups stratified by the 172-gene fibroblast–stemness signature. **C**: overall survival (OS), **D**: recurrence-free survival (RFS), **E**: disease-free survival (DFS)/disease-specific survival (DSS).

To investigate how fibroblast–tumor cell ligand–receptor interactions regulate tumor cells within the SOX9-mutant microenvironment, we performed gene set enrichment analysis (GSEA) comparing Scissor^+^ and Scissor^–^ populations. Scissor^+^ tumor cells exhibited significant upregulation of cell cycle, stemness, and proliferation-associated signaling pathways (Fig. 4A). Moreover, analysis of the C8 cell type gene signature revealed significant enrichment of stem cell, differentiated stem cell, and transit-amplifying cell-associated genes in Scissor^+^ tumor cells (Fig. 4B). Integrating these findings with our cell–cell communication analysis, we propose that aberrant ligand–receptor signaling between fibroblasts and tumor cells in the SOX9-mutant microenvironment promotes stemness by activating these pathways while concurrently enhancing stem-like properties in tumor cells.

### Prognostic value of a fibroblast–stemness gene signature in CRC

Stemness and fibroblast features are established determinants of therapeutic response and clinical outcomes across diverse cancer types [27–29]. We integrated stemness-related gene sets from the CancerSEA database with differentially expressed fibroblast ligands (COL1A1/2, COL6A1/2/3, FN1) identified by CellChat to define a 172-gene signature. Its prognostic value was then evaluated in an independent CRC cohort with clinical survival data. Gene signature scores were computed per patient using GSVA, and patients were stratified into high-score and low-score groups based on median cutoffs. Kaplan–Meier analysis revealed that high-score patients had significantly poorer outcomes, with shorter overall survival (OS) (GSE17538: *P* = 0.026; GSE29621: *P* = 0.0055; Fig. 4C), recurrence-free survival (RFS) (GSE87211: *P* = 0.028; GSE38832: *P* = 0.048; Fig. 4D), and disease-free survival (DFS)/disease-specific survival (DSS) (GSE17538-DSS: *P* = 0.031; GSE161158-DFS: *P* = 0.0059; Fig. 4E). These findings demonstrate that this gene signature is a robust predictor of disease outcomes in CRC patients.

### COL1A1–CD44 interaction enhances stemness in SOX9 truncated mutant colon cancer cells

**Fig. 5:**
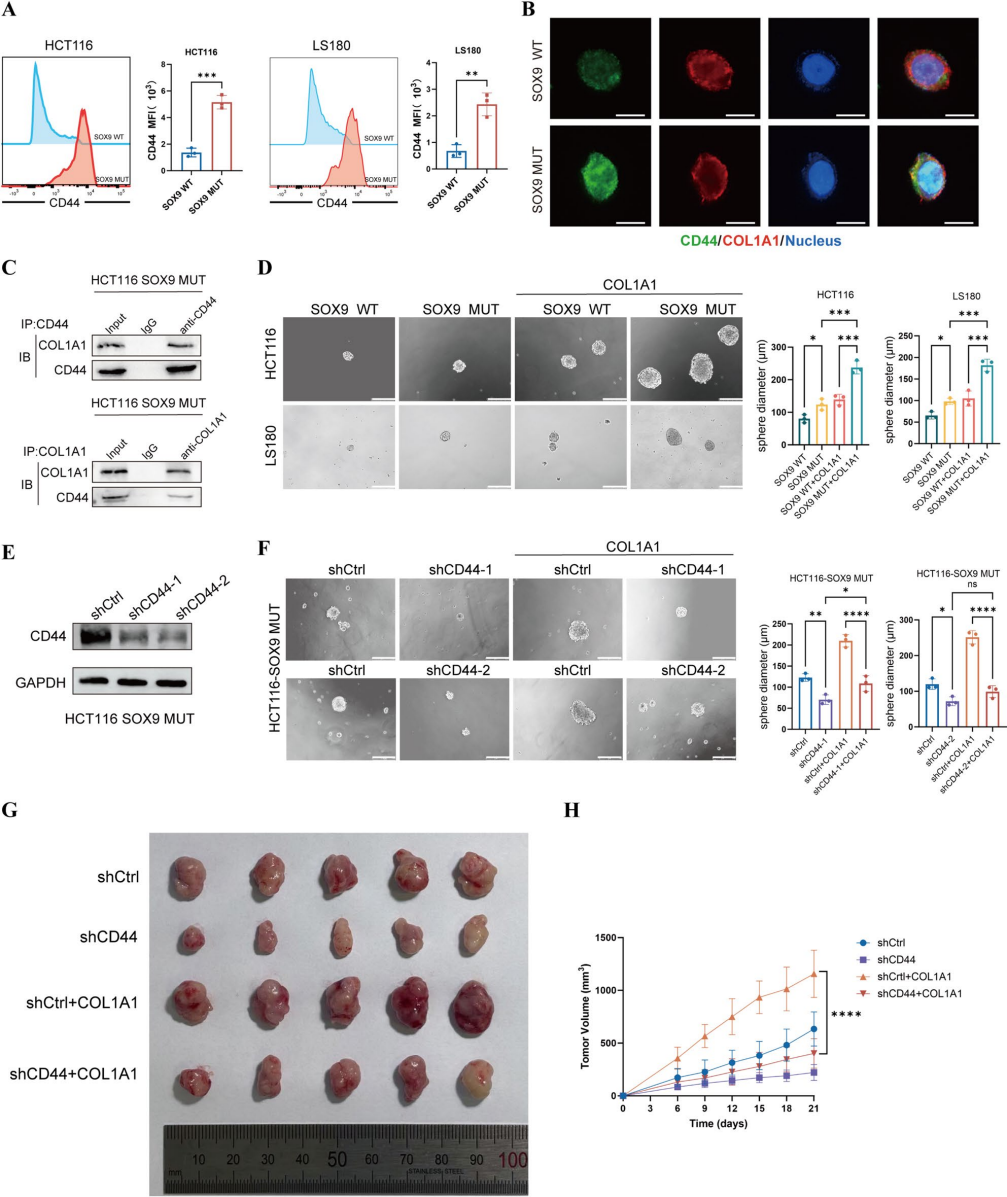
COL1A1–CD44 interaction enhances stemness in SOX9 truncated mutant colon cancer cells. **A** Flow cytometric analysis of CD44 expression in HCT116 cells (left panel) and LS180 cells (right panel) harboring wild-type (WT, blue) or mutant (MUT, red) SOX9. Data are presented as mean ± SEM (*n* = 3). **B** SOX9 WT and SOX9 MUT HCT116 cells were incubated with Biotin-COL1A1 at 4 °C, followed by staining with CD44 (green) and Biotin-COL1A1(red) to examine the colocalization. Scale bar: 10 μm. **C** Co-IP analysis showing an association between CD44 and COL1A1 under extracellular COL1A1 stimulation conditions. HCT116 SOX9 MUT cells were treated with recombinant human COL1A1 protein, lysed, and subjected to immunoprecipitation with anti-CD44 or anti-COL1A1 antibody, followed by immunoblotting with anti-CD44 and anti-COL1A1 antibodies. Whole-cell lysates were analyzed as input controls. Full-length gels are presented in Supplementary Fig. 4. **D** Representative images of sphere formation by HCT116 and LS180 cells (harboring WT or MUT SOX9) following treatment with control or COL1A1 (10 µg/ml). Scale bar: 250 μm. Data are presented as mean ± SEM (*n* = 3). **E** Relative level of CD44 expression in HCT116-SOX9 MUT cells expressing shCtrl or shCD44-1/2. Full-length gels are presented in Supplementary Fig. 4. **F** Representative images of sphere formation by HCT116-SOX9 MUT cells (harboring shCtrl or shCD44) following treatment with control or COL1A1 (10 µg/ml). Scale bar: 250 μm. Data are presented as mean ± SEM (*n* = 3). **G** Gross images of tumors dissected from immunocompromised mice 21 days after subcutaneous implantation of HCT116-SOX9 MUT cells (groups: shCtrl, shCD44, shCtrl+COL1A1, shCD44 + COL1A1). **H** Quantification of tumor volume (measured every 3 days over 21 days) in each experimental group. Data are presented as mean ± SEM (*n* = 5). **A**-**H** An unpaired Student’s t-test or one-way ANOVA or a two-way repeated-measures ANOVA followed by multiple comparisons tests was used to evaluate statistical significance (ns: *P* > 0.05, *: *P* < 0.05, **: *P* < 0.01, ***: *P* < 0.001, ****: *P* < 0.0001)

To validate the role of the COL1A1–CD44 axis in promoting stemness in SOX9-mutant tumor cells, we first examined the expression of CD44 and its interaction with COL1A1 in the SOX9 mutant background. Flow cytometry revealed that compared to SOX9 WT, CD44 expression was significantly upregulated in both cell lines following SOX9 mutation (Fig. 5A). Immunofluorescence experiments further confirmed distinct colocalization between COL1A1 and CD44 in the SOX9 MUT group (Fig. 5B). Co-immunoprecipitation (Co-IP) analysis of SOX9 MUT cells treated with recombinant human COL1A1 demonstrated that CD44 was associated with COL1A1 under extracellular COL1A1 stimulation conditions (Fig. 5C). Subsequently, to elucidate the impact of COL1A1–CD44 interactions on tumor cell self-renewal and stemness, we conducted tumorsphere formation assays. Results showed that COL1A1 treatment markedly enhanced the stemness of both SOX9 MUT cell lines, as evidenced by a significant increase in tumorsphere diameter (Fig. 5D). Conversely, in HCT116-SOX9 MUT cells, knockdown of CD44 using specific short hairpin RNAs (shCD44-1 and shCD44-2) significantly abrogated the COL1A1-induced promotion of tumorsphere formation (Fig. 5E-F). These findings indicate that the COL1A1–CD44 interaction enhances the self-renewal capacity of SOX9 MUT cells.

To further investigate the regulatory role of the COL1A1–CD44 interaction in CRC initiation, we subcutaneously implanted HCT116-SOX9 MUT cells into immunocompromised mice either alone, following CD44 knockdown, or after in vitro co-culture with COL1A1. Results at 21 days post-injection showed significantly reduced tumor growth in shCD44 + COL1A1 nude mice compared to shCtrl+COL1A1 mice, while shCtrl+COL1A1 mice exhibited markedly enhanced tumor formation relative to shCtrl mice (Fig. 5G-H). In summary, COL1A1 partially promotes the self-renewal and in vivo tumorigenicity of the SOX9 truncated mutant in CRC through its interaction with CD44.

## Discussion

CRC constitutes the third most common malignancy worldwide and a leading cause of cancer-related mortality. Molecular studies have revealed that CRC initiation and progression arise from concerted aberrations across multiple genes and pathways, where the interplay between cancer cell mutations and the TME serves as a key driver of malignancy [1, 3, 4]. SOX9 is a pivotal regulator of intestinal development, epithelial homeostasis, and stem cell function that maintains intestinal epithelial stem cell identity and tissue integrity. Previous studies demonstrated that SOX9 plays a context-dependent dual role in solid tumors, with its mutations or dysregulated expression linked to stemness maintenance, epithelial-mesenchymal transition, and therapeutic resistance [5, 6]. However, conventional bulk RNA-seq can only identify transcriptomic alterations in SOX9-mutant CRC, but failed to resolve cellular heterogeneity masked by averaged signals from mixed cell populations. It lacked the ability to delineate, at single-cell resolution, the cell type-specific molecular programs and intercellular communication networks shaped by SOX9 mutations, thereby limiting further understanding of the mechanisms underlying disease progression. To overcome this limitation, we integrated scRNA-seq data from 23 CRC samples (GSE132465) with SOX9 mutation status, which was obtained from the TCGA-COAD cohort, using the Scissor algorithm, and for the first time precisely identified cell subpopulations specifically associated with SOX9 mutations (Scissor⁺ cells).

CellChat-based cell communication analysis revealed that, compared with SOX9 wild-type tumors, the COL1A1–CD44 signaling axis was markedly upregulated in the TME of SOX9-mutant tumors. Notably, signaling interactions between fibroblasts and tumor cells were most prominently enhanced, becoming the dominant intercellular communication pathway. Tumorsphere formation assays demonstrated that COL1A1 stimulation promoted sphere formation in SOX9 MUT cells, whereas CD44 knockdown effectively abrogated this stemness promoting effect. In vivo xenograft models further supported these findings, showing that inhibition of CD44 attenuates the tumor promoting effects of COL1A1. Collectively, these results indicate that SOX9 mutations enhance tumor stemness through the COL1A1–CD44 axis mediated fibroblast–tumor cell interactions.

Importantly, the upregulation of this signaling axis is not a universal feature across all pathological contexts of CRC, but is specifically pronounced in the setting of SOX9 mutations. This provides a molecular explanation for clinical observations: although SOX9 mutations occur in only approximately 5%–10% of CRC patients, they are significantly associated with increased tumor aggressiveness and poor prognosis [30–32]. Previous studies have largely attributed such phenotypes to fibroblast activation or collagen deposition. For instance, in colorectal cancer liver metastasis, myofibroblast-like cancer associated fibroblasts secrete extracellular matrix components such as fibronectin, which bind to CD44 on tumor cells to maintain stemness and metastatic potential [33]. In contrast, our findings suggest that SOX9-mutant tumors themselves can drive phenotypic and functional reprogramming of tumor cells, characterized by a stem-like transcriptional state, enrichment of stemness related pathways, and marked upregulation of the CD44 receptor, suggesting a heightened capacity to engage extracellular matrix-derived ligands. Meanwhile, SOX9-mutant tumors not only alter tumor intrinsic signaling but also remodel the stromal microenvironment: although the overall abundance of fibroblasts remains largely unchanged, their transcriptional programs are reprogrammed, with significant upregulation of extracellular matrix related genes, particularly COL1A1. Thus, SOX9-mutant tumors primarily reprogram fibroblast function rather than regulating their abundance. Previous studies have shown that increased deposition is a hallmark of extracellular matrix remodeling and is often closely associated with elevated tissue stiffness [34, 35]. Accumulating evidence indicates that a stiff extracellular matrix can induce cancer stem cell properties via mechanotransduction pathways, promoting CD44 expression in tumor cells. CD44 has been reported to function as a mechanosensitive receptor that senses and transduces matrix stiffness signals, activating downstream pathways such as FAK/Src-PI3K-Akt-mTOR and Hippo-YAP, thereby forming a positive feedback loop between matrix signaling and stemness amplification [35–38].

From the clinical perspective, immunohistochemical analyses confirmed significant upregulation of COL1A1 and CD44 expression in tumor tissues from patients with SOX9 mutations, with evident spatial co-localization. Furthermore, we constructed a fibroblast–stemness gene signature model integrating extracellular matrix ligands and stemness associated genes, which robustly predicted patient survival across multiple independent CRC cohorts. By capturing both tumor intrinsic features and microenvironmental regulatory effects, this signature provides a more comprehensive prognostic framework compared with traditional single gene biomarkers [39–41]. More importantly, targeting the COL1A1–CD44 axis and its downstream signaling pathways may represent a promising therapeutic strategy for SOX9-mutant CRC, particularly for patients with high stemness and poor prognosis.

In summary, our study demonstrates that SOX9-mutant tumors reshape the TME by reprogramming interactions between fibroblasts and tumor cells and activating COL1A1–CD44 mediated stemness programs, thereby promoting the maintenance of tumor stemness and driving CRC progression. Although our findings establish an association between SOX9 mutations and fibroblast activation, the current evidence suggests that fibroblast phenotypic changes are more likely secondary effects resulting from SOX9 mutation-driven reprogramming of tumor cell activity. A direct causal relationship between SOX9 mutations, fibroblast phenotypic alterations, and mechanical stress remains to be fully established. Further studies are warranted to elucidate the molecular mechanisms linking SOX9 mutations, tumor matrix remodeling, increased mechanical stress, and the maintenance of tumor stemness.

## Supplementary Information

Supplementary Material 1.

## Data Availability

All data used in this study are publicly available. The scRNA-seq dataset was obtained from the Gene Expression Omnibus (GEO) database (accession number: GSE132465; https://www.ncbi.nlm.nih.gov/geo/). The bulk RNA-seq datasets and corresponding clinical information were retrieved from The Cancer Genome Atlas (TCGA) and GEO databases (accession numbers: GSE17538, GSE29621, GSE87211, GSE38832, and GSE161158). These datasets are freely accessible for download from the respective repositories.
