# Supplementary material for: Single-cell transcriptome analysis reveals SOX9 mutation-driven tumor stemness and microenvironment remodeling through the COL1A1–CD44 axis in colorectal cancer
Source: BMC Cancer. 2026 May 30;26:921. doi: 10.1186/s12885-026-16248-z (PMC13430886; doi:10.1186/s12885-026-16248-z)
Supplement: Supplementary file 1 — Supplementary Material 1. [file 12885_2026_16248_MOESM1_ESM.pdf]

Supplementary Material

Supplementary Table 1. Key Resources

**Reagents or Resource**	**Source**	**Identifier**
Alpha smooth muscle actin Polyclonal antibody	Proteintech	14395-1-AP
COL1A1 (E8F4L) Rabbit Monoclonal Antibody	Cell Signaling Technology	72026
CD44 (E7K2Y) Rabbit Monoclonal Antibody	Cell Signaling Technology	37259
Anti-SOX9 antibody	Abcam	ab185966
CD44 Monoclonal Antibody	Invitrogen	14-0441-82
APC anti-mouse/human CD44 Antibody	Biolegend	103011
Fixable Viability Dye	eBioscience	65-0865-14
Biotin-conjugated type I collagen	Invitrogen	PA1-28530
Biotin Polyclonal Antibody	Invitrogen	31852
Donkey anti-Mouse IgG (H+L) Highly Cross-Adsorbed Secondary Antibody, Alexa Fluor™ 488	Invitrogen	A-21202
Donkey anti-Rabbit IgG (H+L) ReadyProbes™ Secondary Antibody, Alexa Fluor™ 594	Invitrogen	R37119
Anti-Collagen I antibody [EPR24331-53]	Abcam	ab270993
Anti-CD44 antibody	Abcam	ab157107

Supplementary Table 2. Complete list of genes included in the 172-gene fibroblast–stemness signature

Fibroblast Ligands	Stemness genes form CancerSEA						
COL1A1	AFMID	CFTR	FOXA2	IKZF1	NELL2	RGMB	TFDP2
COL1A2	AFP	CHD7	FOXA3	IRX3	NFE2	RNF43	TFRC
COL6A1	ANPEP	CORO1C	FOXG1	ITGAM	NFIA	RUNX1	THY1
COL6A2	APC	CXCL2	GATA1	KDR	NFIB	SET	TNFAIP8L1
COL6A3	AQP1	DBX1	GATA2	KIT	NKX2-5	SLC12A2	TOX3
FN1	ASCL1	DBX2	GATA3	KLK10	NODAL	SMAD2	TRA2A
	ASCL2	DNMT3A	GATA4	KRT14	NT5E	SMOC2	TSPAN6
	AXIN2	DPP4	GFAP	LATS2	OLFM4	SOX1	UGT8
	AZGP1	EBF1	GFI1	LGR5	OPHN1	SOX11	VEGFA
	BAZ2B	EEF1A1	GLI1	LOH12CR2	ORC6	SOX17	ZBTB8A
	BMI1	EGFR	GPC3	LRIG1	PABPC1	SOX2	ZNF793
	BOC	EIF4B	GPM6A	LY6D	PAX6	SOX3	ABCG2
	C6orf62	EMX1	GPSM2	LYZ	POU5F1	SOX4	ALDH1A1
	CA2	EMX2	GPX2	MALAT1	PROM1	SOX9	ALDH1A3
	CAMK2N1	ENG	H19	MBOAT1	PTK7	SPDYE1	DNER
	CCL5	EPHB2	H3F3B	MESP1	PTMA	SPDYE5	MET
	CCND2	ETS2	HACD3	MESP2	PTPRC	SPHKAP	
	CD200	ETV1	HAPLN1	METTL3	PTPRG	SRGAP2C	
	CD24	EVI2A	HEPN1	MLLT10	PTPRO	STMN1	
	CD33	EZH2	HES1	MME	PTPRS	STMN2	
	CD38	FABP7	HNRNPH1	MYB	PTPRZ1	TAL1	
	CD44	FAM84A	HNRNPL	MYC	QPCTL	TATDN3	
	CDCA7	FBLIM1	HOPX	NANOG	RAB42	TCF12	
	CDK6	FBXO27	ICAM1	NCAM1	RAMP2-AS1	TCF4	
	CEBPA	FERMT1	IDH1	NEK5	RBM6	TDGF1	

Figure S1. The single-cell landscape of CRC patients

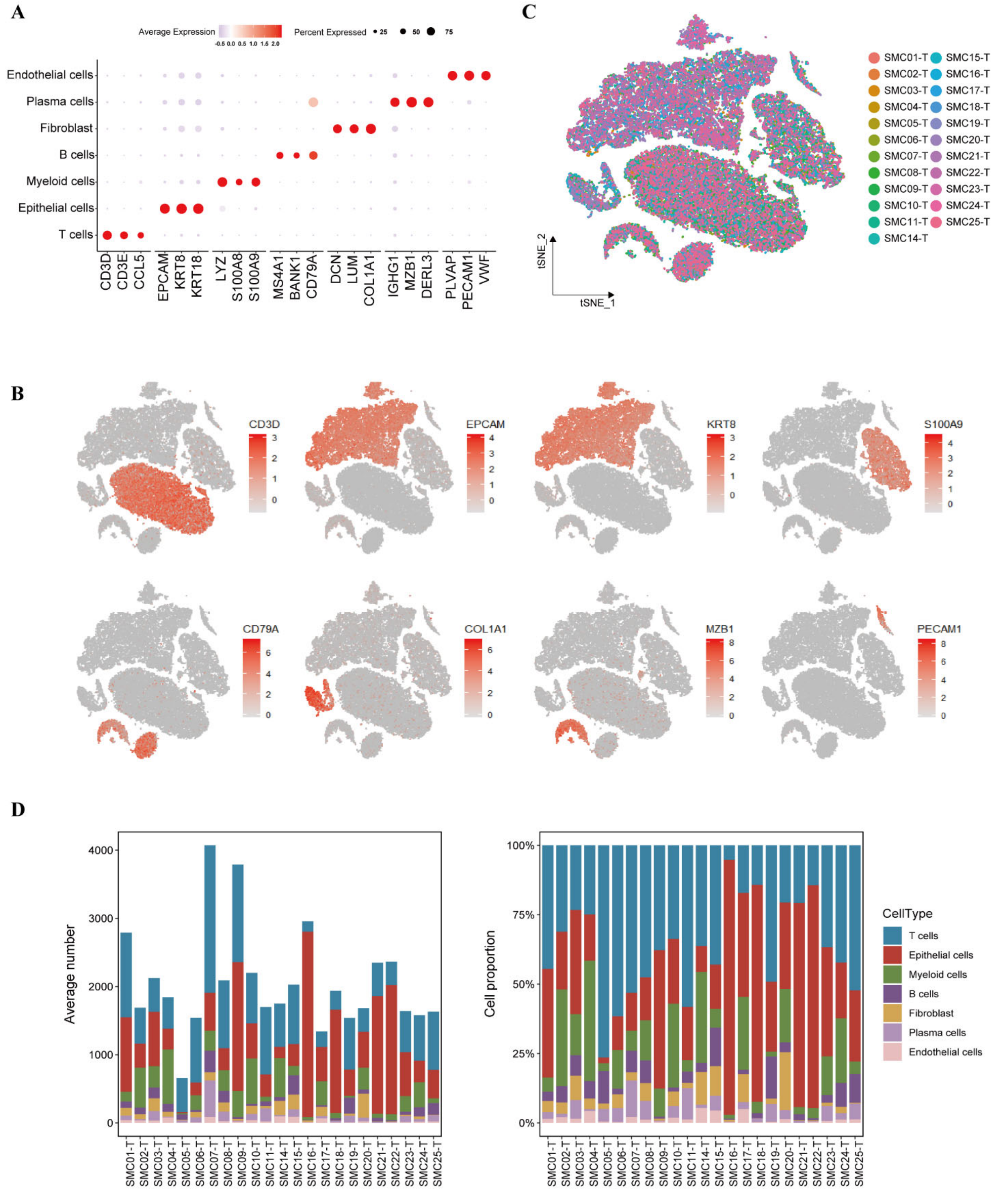

(A) Dotplot shows the canonical markers across distinct cell types. **(B)** Feature plots present the normalized expression levels of canonical marker genes. **(C)** t-SNE projection of clusters from 23 samples. **(D)** Characterization of the cell type abundance and proportions identified in each sample.

Figure S2. Analysis of differential intercellular communication between Scissor^{+} and Scissor^{-} cells

A

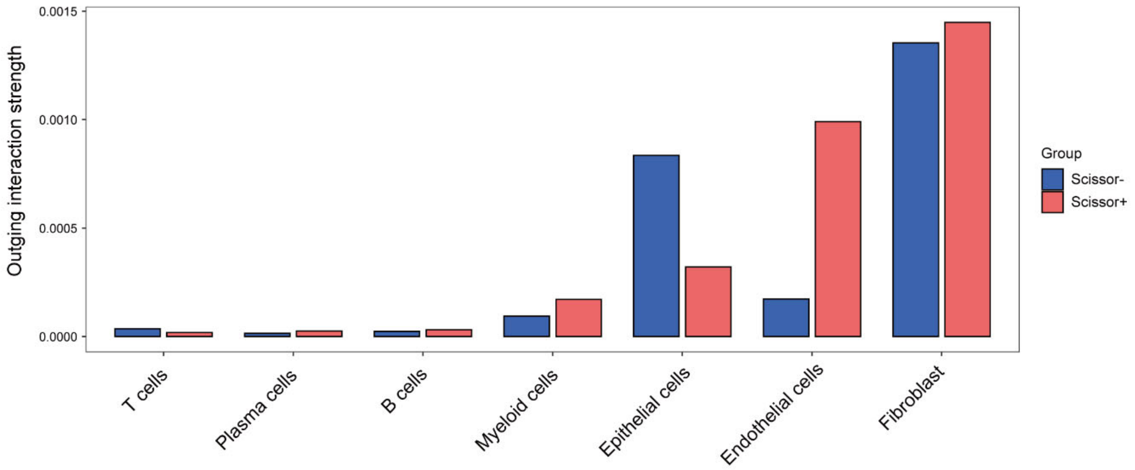

Outging interaction strength
0.0015
0.0010
0.0005
0.0000
T cells
Plasma cells
B cells
Myeloid cells
Epithelial cells
Endothelial cells
Fibroblast
Group
Scissor-
Scissor+

B

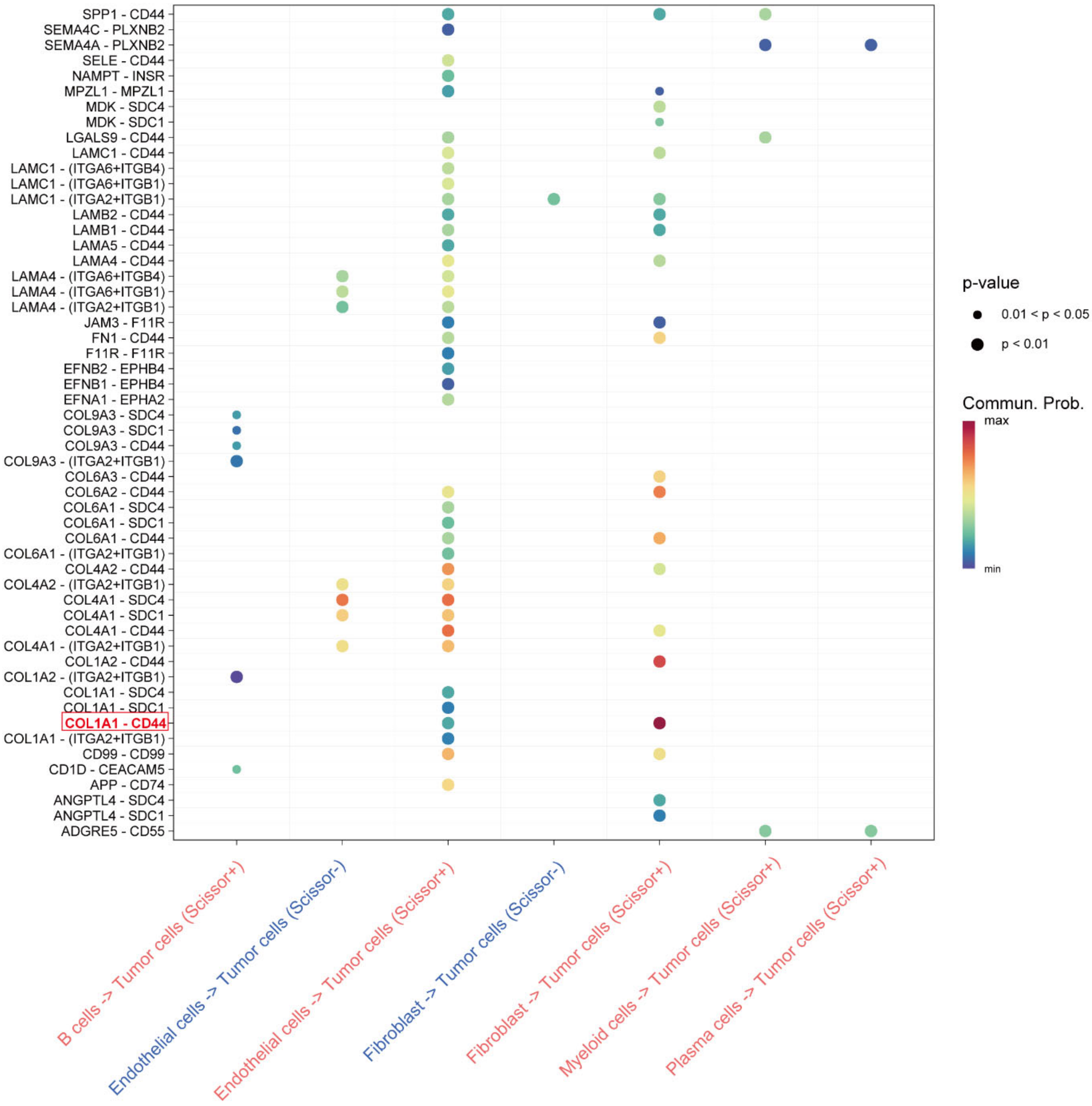

SPP1 - CD44
SEMA4C - PLXNB2
SEMA4A - PLXNB2
SELE - CD44
NAMPT - INSR
MPZL1 - MPZL1
MDK - SDC4
MDK - SDC1
LGALS9 - CD44
LAMC1 - CD44
LAMC1 - (ITGA6+ITGB4)
LAMC1 - (ITGA6+ITGB1)
LAMC1 - (ITGA2+ITGB1)
LAMB2 - CD44
LAMB1 - CD44
LAMA5 - CD44
LAMA4 - CD44
LAMA4 - (ITGA6+ITGB4)
LAMA4 - (ITGA6+ITGB1)
LAMA4 - (ITGA2+ITGB1)
JAM3 - F11R
FN1 - CD44
F11R - F11R
EFNB2 - EPHB4
EFNB1 - EPHB4
EFNA1 - EPHA2
COL9A3 - SDC4
COL9A3 - SDC1
COL9A3 - CD44
COL9A3 - (ITGA2+ITGB1)
COL6A3 - CD44
COL6A2 - CD44
COL6A1 - SDC4
COL6A1 - SDC1
COL6A1 - CD44
COL6A1 - (ITGA2+ITGB1)
COL4A2 - CD44
COL4A2 - (ITGA2+ITGB1)
COL4A1 - SDC4
COL4A1 - SDC1
COL4A1 - CD44
COL4A1 - (ITGA2+ITGB1)
COL1A2 - CD44
COL1A2 - (ITGA2+ITGB1)
COL1A1 - SDC4
COL1A1 - SDC1
COL1A1 - CD44
COL1A1 - (ITGA2+ITGB1)
CD99 - CD99
CD1D - CEACAM5
APP - CD74
ANGPTL4 - SDC4
ANGPTL4 - SDC1
ADGRE5 - CD55
B cells -> Tumor cells (Scissor+)
Endothelial cells -> Tumor cells (Scissor-)
Endothelial cells -> Tumor cells (Scissor+)
Fibroblast -> Tumor cells (Scissor-)
Fibroblast -> Tumor cells (Scissor+)
Myeloid cells -> Tumor cells (Scissor+)
Plasma cells -> Tumor cells (Scissor+)
p-value
0.01 < p < 0.05
p < 0.01
Commun. Prob.
max
min

(A) Bar chart depicting outgoing interaction strength of indicated cell types in Scissor^{-} (blue) and Scissor^{+} (red) groups. **(B)** Dotplot of differential ligand-receptor interactions between Scissor^{+} and Scissor^{-} cells, where dot color indicates interaction probability and size denotes significance.

Figure S3. SOX9 mutation remodels fibroblast-tumor cell communication and upregulates the COL1A1-CD44 ligand-receptor axis

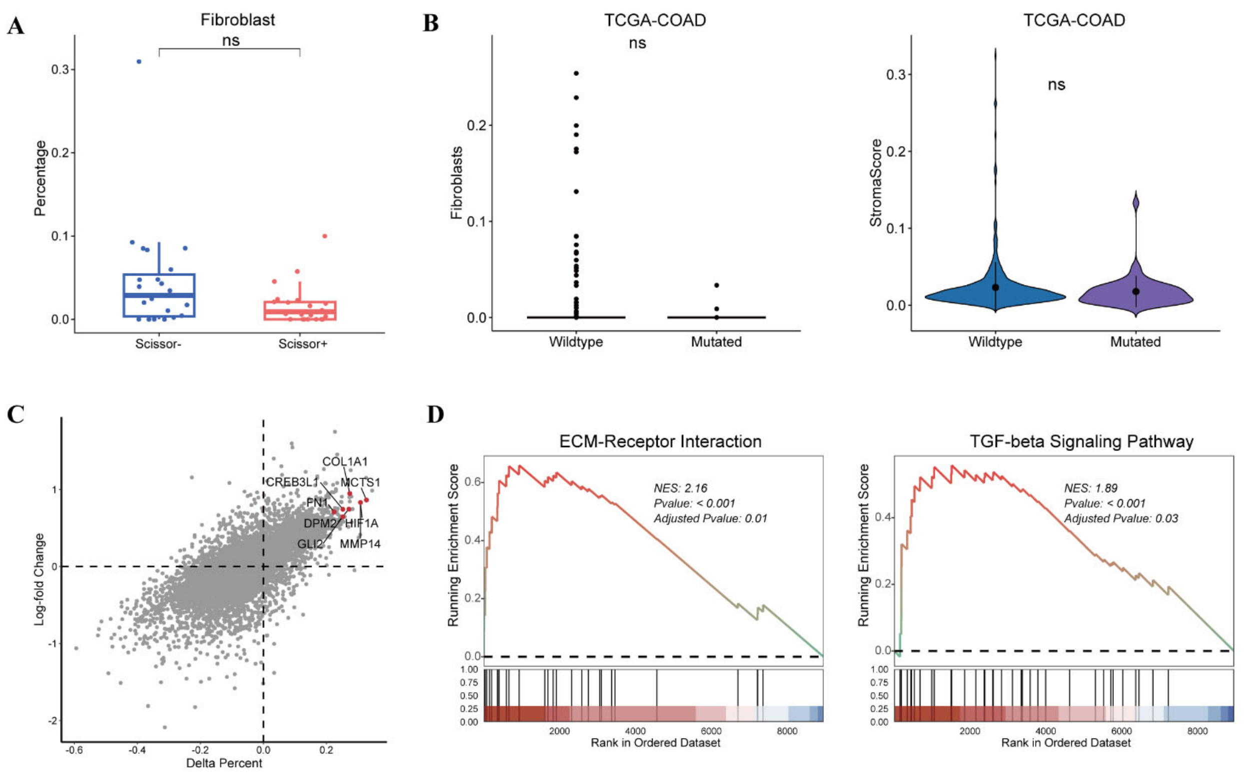

(A) Box plot showing the proportion of fibroblasts among total Scissor^{-} and Scissor^{+} cells per patient. **(B)** Violin plots comparing fibroblast proportions (left) and stroma score (right) between SOX9 wild-type and SOX9-mutant patients in the TCGA-COAD cohort, analyzed using the xCell algorithm. **(C)** Volcano plot showing differentially expressed genes (DEGs) between Scissor^{-} and Scissor^{+} fibroblasts. **(D)** GSEA enrichment plots demonstrating significant upregulation of ECM-receptor interaction and

TGF-β signaling pathway. All pathways showed adjusted P-values < 0.001. (A-B) An unpaired Student's t-test was used to evaluate statistical significance(ns:P > 0.05).

Figure S4. Uncropped gels for Western Blots in Figure 5

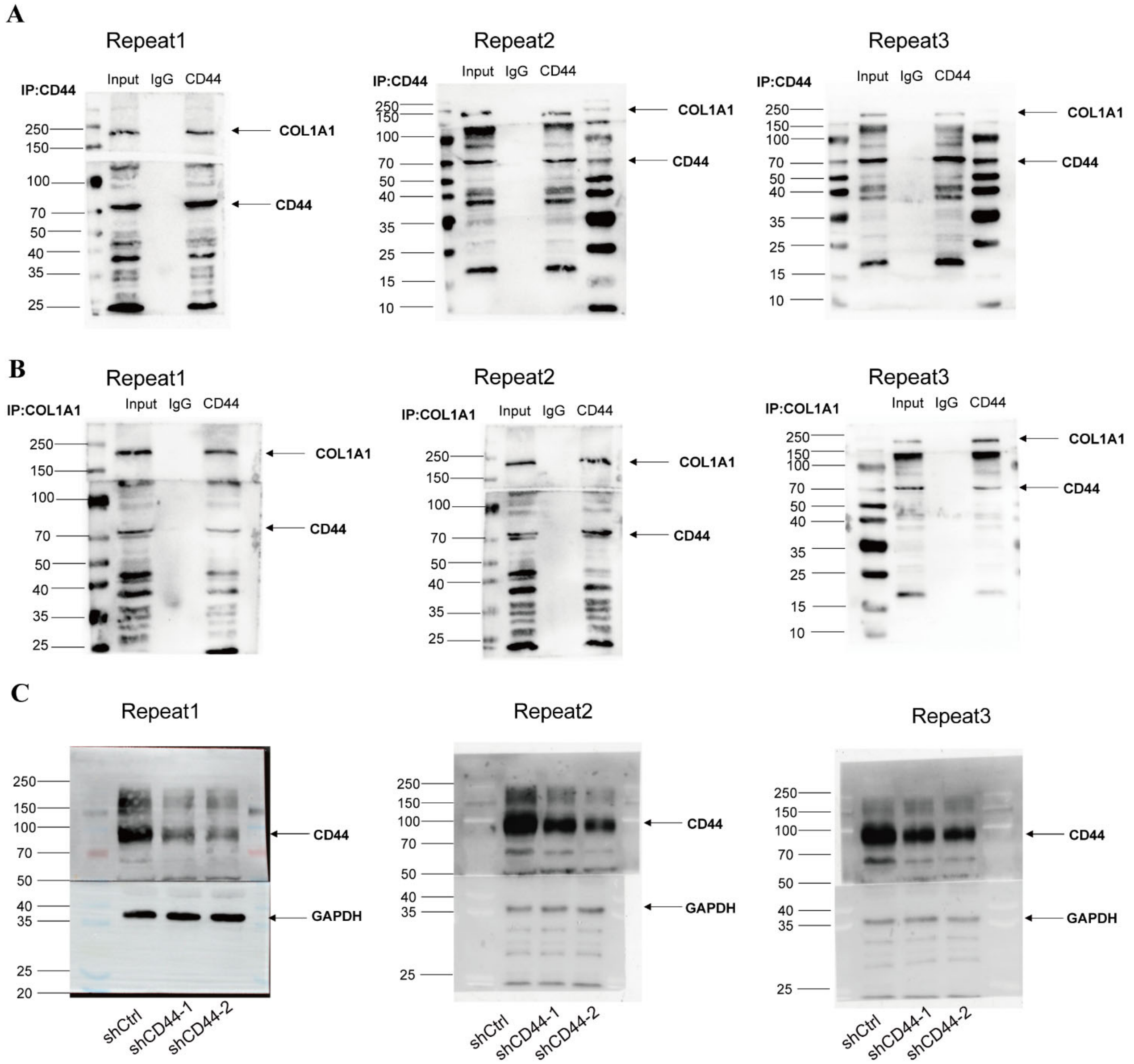

(A-B) Uncropped gels for IPs and IBs for Figure 5C. **(C)** Uncropped gels for Western Blots for Figure 5E.
